# Supplementary material for: Constitutive hippocampal cholesterol loss underlies poor cognition in old rodents
Source: EMBO Mol Med. 2014 May 30;6(7):902–17. doi: 10.15252/emmm.201303711 (PMC4119354; doi:10.15252/emmm.201303711)
Supplement: Supplementary file 1 — Supplementary Figure S1 [file emmm0006-0902-SD1.pdf]

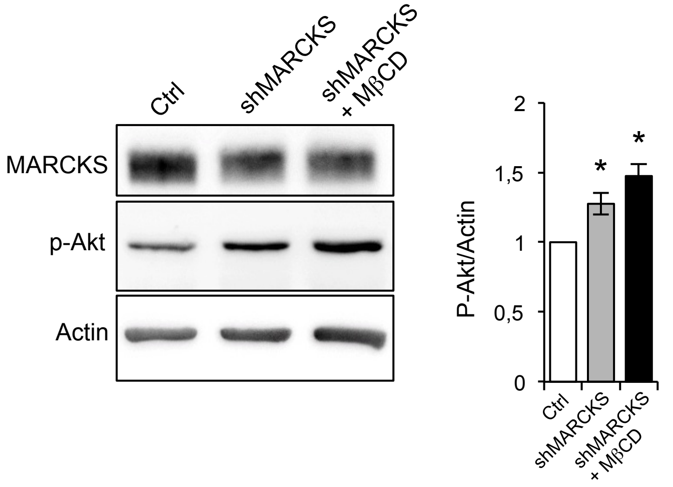

Supplementary Figure S1. MARCKS knock down results in increased p-Akt levels and cholesterol decrease potentiate this effect.
p-Akt was measured by Western blot in 15DIV neurons infected with lentiviral particles that express shRNA designed to knock down MARCKS (shMARCKS). MARCKS knock down resulted in increased p-Akt levels compared to controls. Cholesterol extraction from shMARCKS treated neurons (shMARCKS + MβCD) resulted in a further increase of Akt phosphorylation. The amount of p-Akt (rdative to untreated controls) were: shMARCKS = 1.28 ± 0.054 and shMARCKS + chol = 1.47 ± 0.062 ($p = 0.036$, $n = 3$ different cultures).
