# Supplementary material for: Constitutive hippocampal cholesterol loss underlies poor cognition in old rodents
Source: EMBO Mol Med. 2014 May 30;6(7):902–17. doi: 10.15252/emmm.201303711 (PMC4119354; doi:10.15252/emmm.201303711)

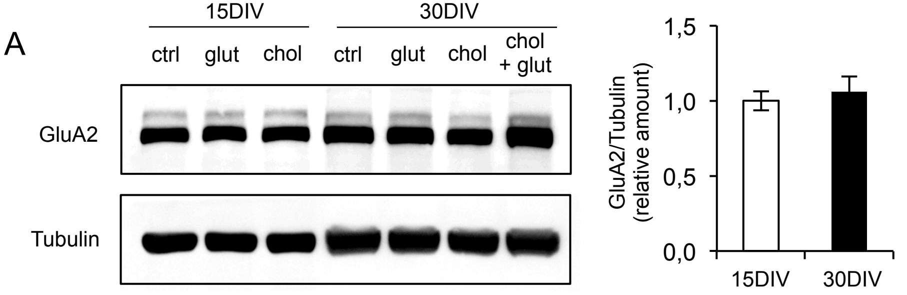

Supplementary Figure S2. The ratio of internalized AMPARs increases after glutamate stimulation in 30DIV cholesterol-replenished neurons.

A) Representative western blot and quantification shows that total GluA2 levels does not change in the different conditions tested in hippocampal neurons in vitro.

B) Fluorescence microscopy images show the levels of surface (red) and internalized (green) GluA2 containing AMPARs in stimulated or non-stimulated 15DIV neurons (left panel). The quantification of fluorescence intensity (internalized/total GluA2) shows that after glutamate stimulation, the ratio of internalized AMPARs increased 26% (glut) compared to non-stimulated (ctrl) neurons (right panel, Control = 1 ± 0.052, glut = 1.26 ± 0.080, p = 0.042, n = 3).

C) The same antibody feeding experiments were used to compare the ratio of internalized GluA2 in untreated or cholesterol replenished 30DIV neurons, both in control conditions and after glutamate stimulation. The plot on the right shows that in cholesterol replenished 30DIV neurons, glutamate stimulation resulted in a significant increase in the ratio of internalized receptors. The value for 30DIV neurons treated with glutamate was statistically similar to that of the controls (Ctrl = 1 ± 0.12, glut = 1.07 ± 0.039; Mann Whitney test, $p^{30\ DIVglu,\ 30\ DIVcontrol}$ = 0.75; n = 4) or the cholesterol-treated cells without glutamate (chol: 1.15 ± 0.03; $p^{30\ DIVglu,\ 30\ DIVchol}$ =0.56; $p^{30\ DIVcontrol,\ 30\ DIVchol}$ = 0.47; n = 4). However, the addition of cholesterol to 30 DIV neurons prior to the glutamate administration yielded a significant increase in the ratio of internalized receptors (1.89 ± 0.19; $p^{30\ DIVcontrol,\ 30\ DIVchol+glu}$ = 0.0027; $p^{30\ DIVglu,\ 30\ DIVchol+glu}$ = 0.0002; n = 4).

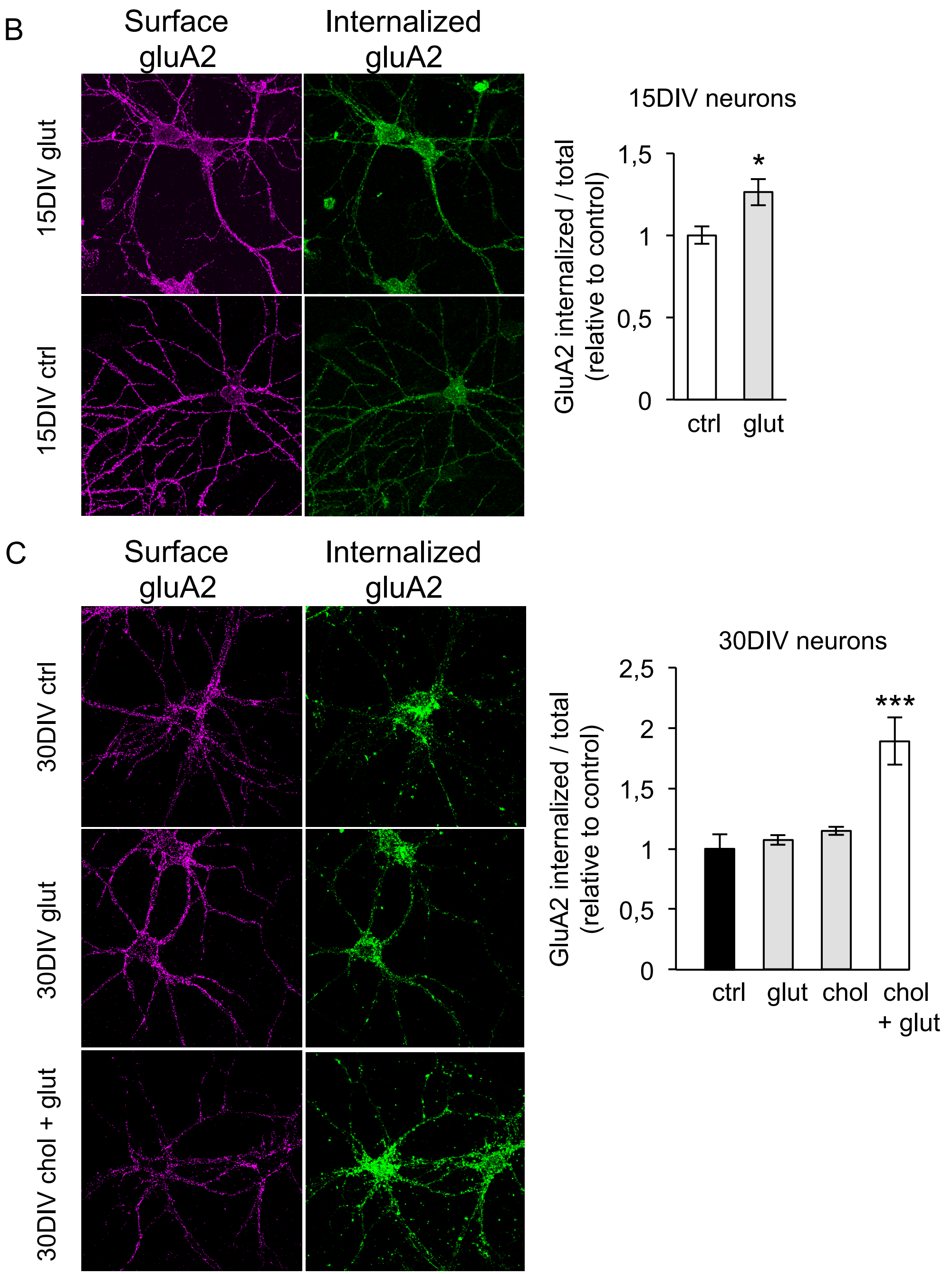

Supplement: Supplementary file 2 — Supplementary Figure S2 [file emmm0006-0902-SD2.pdf]
