# Supplementary material for: Constitutive hippocampal cholesterol loss underlies poor cognition in old rodents
Source: EMBO Mol Med. 2014 May 30;6(7):902–17. doi: 10.15252/emmm.201303711 (PMC4119354; doi:10.15252/emmm.201303711)
Supplement: Supplementary file 3 — Supplementary Figure S3 [file emmm0006-0902-SD3.pdf]

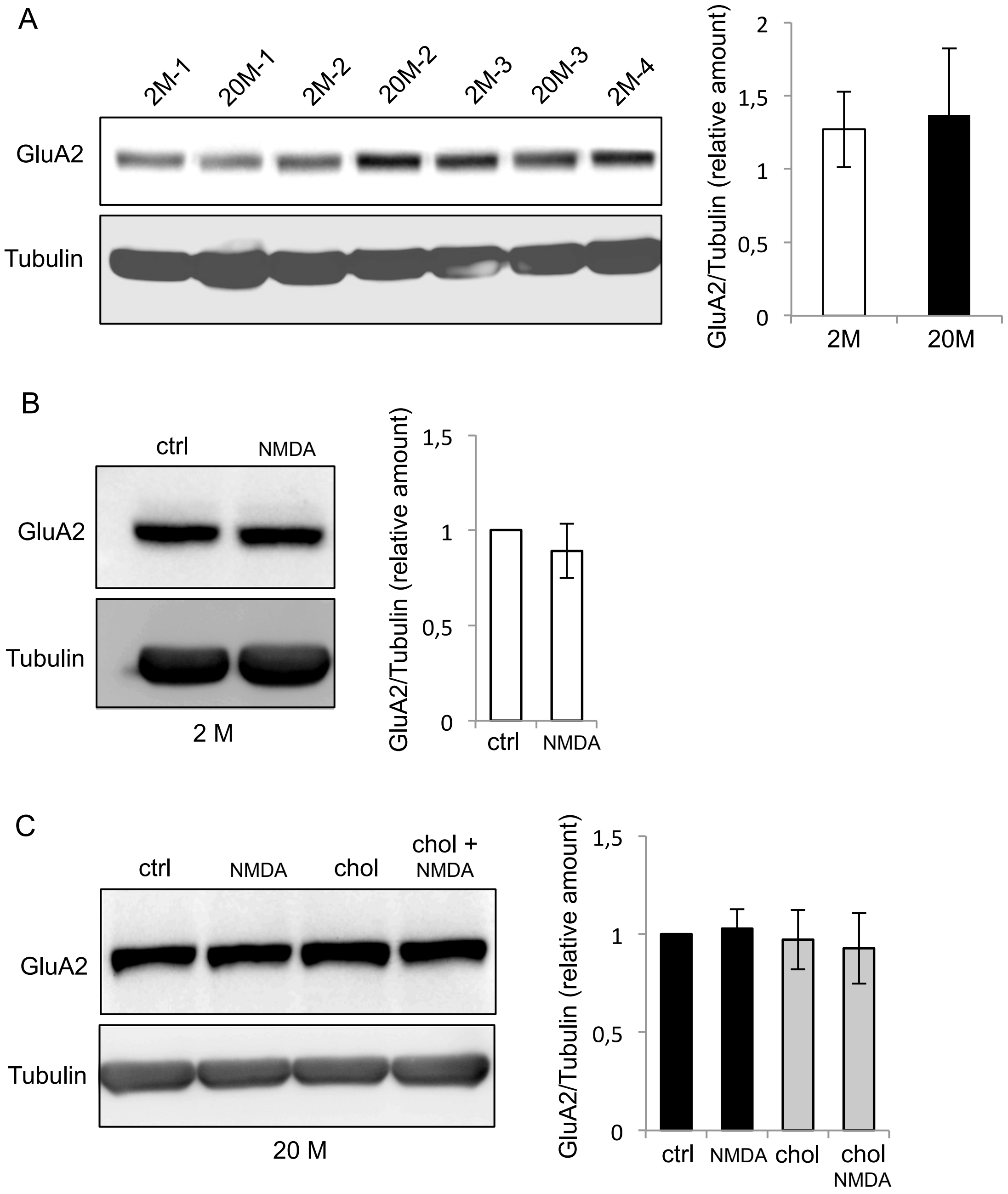

Supplementary Figure S3.

Total levels of GluA2 do not change in the different conditions studied in vivo.

The Western blots (left) and its quantifications (right) show that same levels of total GluA2 are present in: A) 2 month-old (2M) and 20 month-old mice (20M).

B) Hippocampal slices prepared from 2M mice in control conditions (ctrl) or after NMDA stimulation (NMDA).

C) Hippocampal slices prepared from 20M mice in control conditions (ctrl), after NMDA stimulation (NMDA), cholesterol replenishment (chol) and cholesterol replenishment plus NMDA stimulation (chol + NMDA).
