# Supplementary material for: Constitutive hippocampal cholesterol loss underlies poor cognition in old rodents
Source: EMBO Mol Med. 2014 May 30;6(7):902–17. doi: 10.15252/emmm.201303711 (PMC4119354; doi:10.15252/emmm.201303711)
Supplement: Supplementary file 4 — Supplementary Figure S4 [file emmm0006-0902-SD4.pdf]

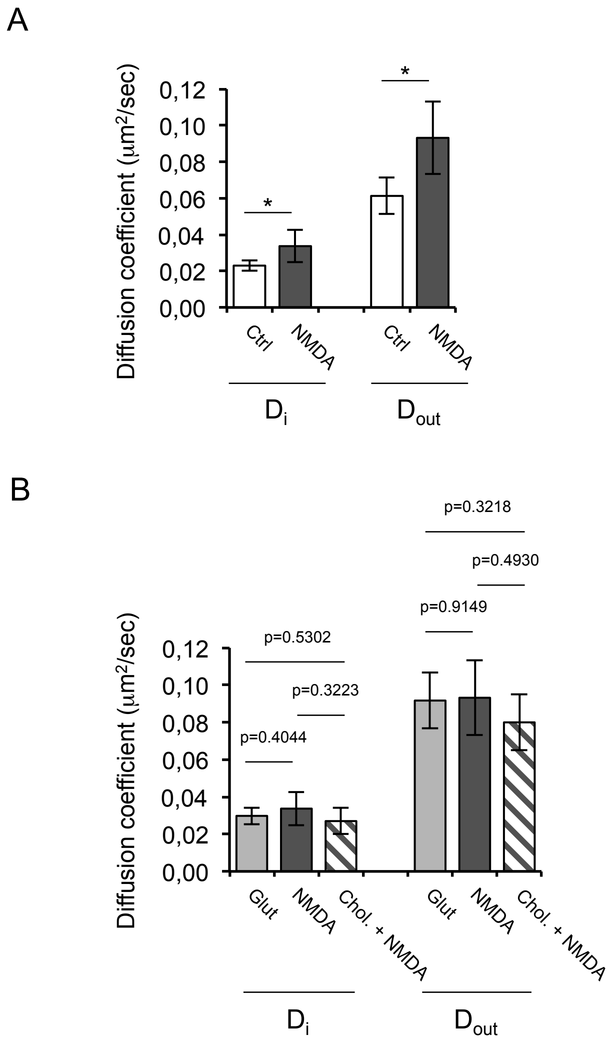

Supplementary Figure S4. No differences in lateral diffusion are observed between NMDA, glutamate or cholesterol + NMDA treated neurons.

A) NMDA stimulation increases the diffusion of synaptic and extra-synaptic AMPARs in 15 DIV. D_i control = 0.023 ± 0.0028 mm^2/s, D_i NMDA = 0.0337 ± 0.009 µm^2/s (p = 0.0339), D_{out} control = 0.0613 ± 0.0099 µm^2/s, D_{out} NMDA = 0.0932 ± 0.02 µm^2/s (p = 0.0496).
B) No differences were observed in the lateral diffusion of synaptic and extra-synaptic AMPARs between glutamate or NMDA stimulated cells. The plot also shows that in control experiments, cholesterol addition to 15 DIV neurons does not affect the lateral diffusion of these receptors after stimulation.
