# Supplementary material for: Constitutive hippocampal cholesterol loss underlies poor cognition in old rodents
Source: EMBO Mol Med. 2014 May 30;6(7):902–17. doi: 10.15252/emmm.201303711 (PMC4119354; doi:10.15252/emmm.201303711)
Supplement: Supplementary file 5 — Supplementary Figure S5 [file emmm0006-0902-SD5.pdf]

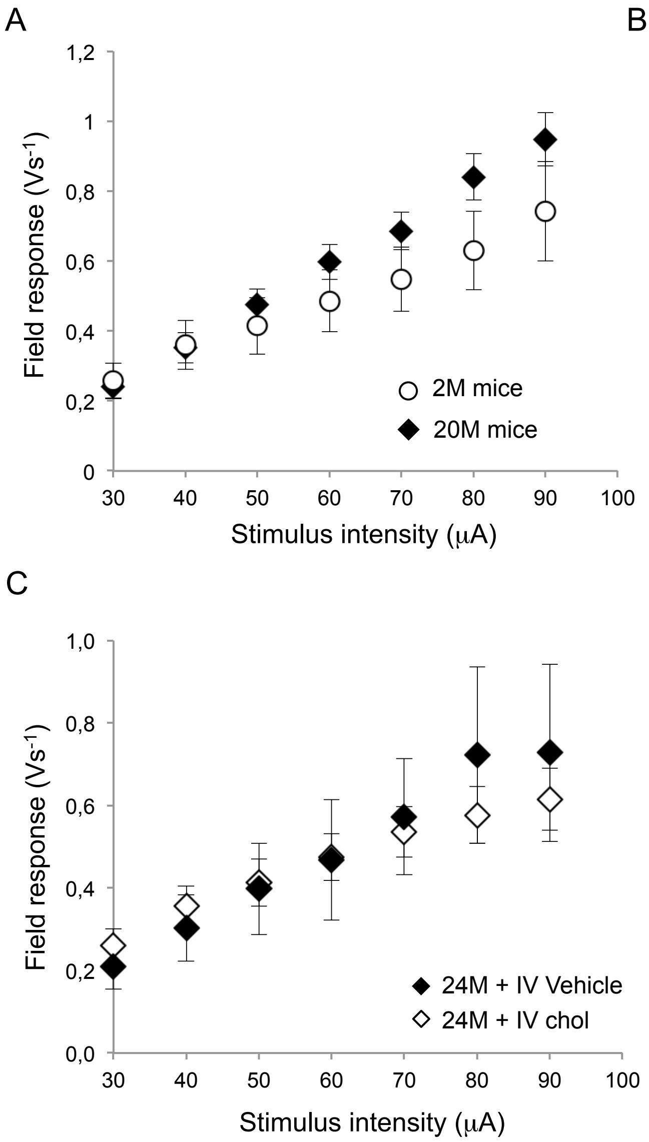

Supplementary Figure S5.

Basal synaptic transmission is not affected by aging, cholesterol depletion or cholesterol perfusion.

The figure shows the input-output curves recorded from A) Hippocampal slices prepared from young (2M) and old (20M) mice, B) Hippocampal slices prepared from middle aged (10M) and middle aged mice depleted of cholesterol (10M + MbCD) and C) Hippocampal sliced obtained from old mice perfused with vehicle (24M +IV vehicle) or cholesterol (24M +IV chol). Two-way Anova analysis indicates that no significant differences were observed between the two conditions analyzed in each case.
