# Supplementary material for: Constitutive hippocampal cholesterol loss underlies poor cognition in old rodents
Source: EMBO Mol Med. 2014 May 30;6(7):902–17. doi: 10.15252/emmm.201303711 (PMC4119354; doi:10.15252/emmm.201303711)
Supplement: Supplementary file 6 — Supplementary Figure S6 [file emmm0006-0902-SD6.pdf]

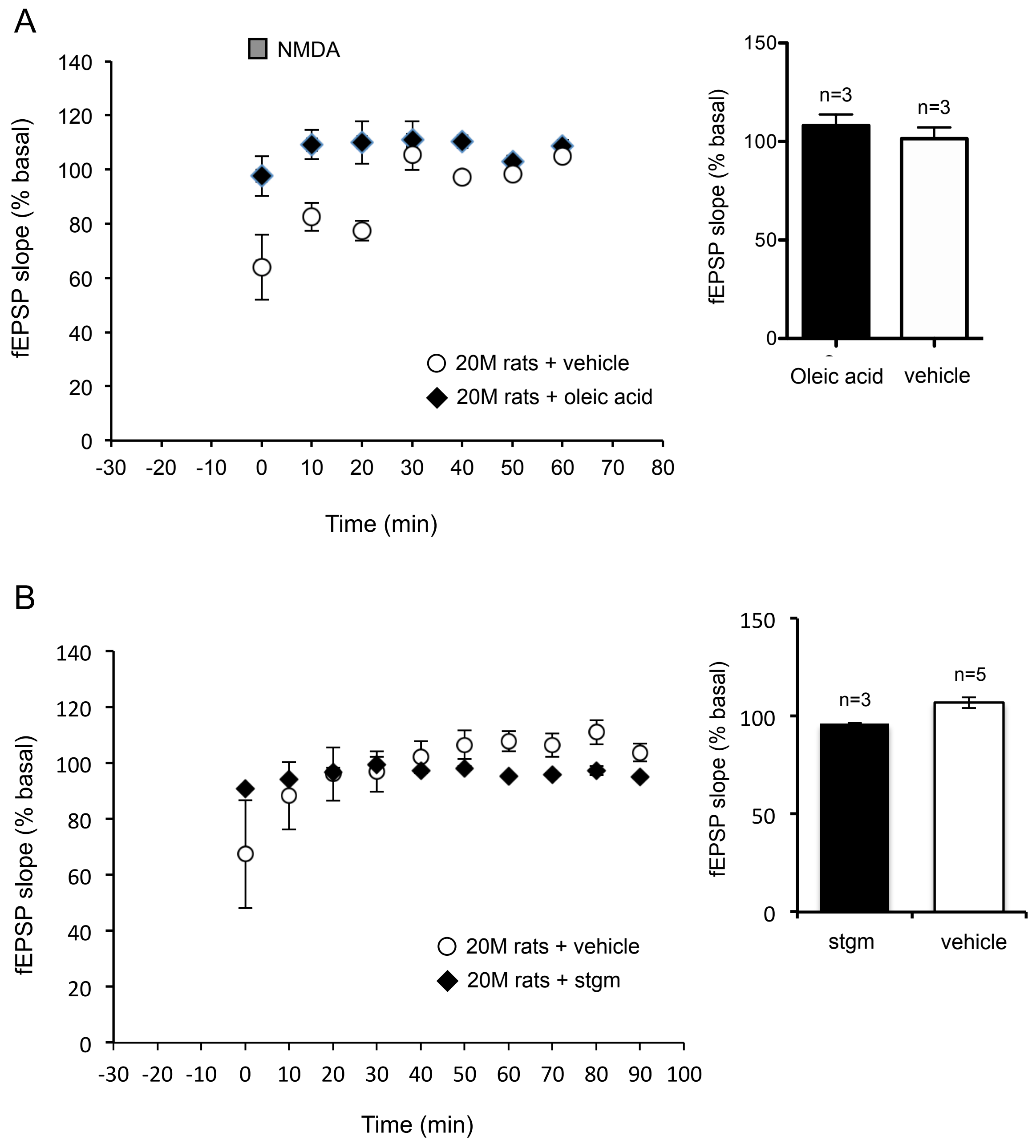

Supplementary Figure S6.

Impaired LTD in old rats could not be restored by injection of another lipid-cyclodextrin complexes.

A) The plot shows the evolution of fEPSPs in vehicle (○) or oleic acid-MbCD injected (◆) 20M Wistar rats following NMDA-inducing LTD protocol.

B) Evolution of fEPSPs in vehicle (○) or stigmasterol-MbCD injected (◆) 20M Wistar rats following NMDA-inducing LTD protocol.

The plots on the left show the average of the fEPSRs obtained during the last 30 min. As can be seen in the figure, impaired LTD in 20-month old (20M) animals could not be restored by injection of either oleic acid-MbCD (oleic acid) or the cholesterol analog, stigmasterol-MbCD complex (stgm). Data are presented as mean ± s.e.m. The number of animals employed is indicated (n) in the bar plots.
