# Supplementary material for: Constitutive hippocampal cholesterol loss underlies poor cognition in old rodents
Source: EMBO Mol Med. 2014 May 30;6(7):902–17. doi: 10.15252/emmm.201303711 (PMC4119354; doi:10.15252/emmm.201303711)
Supplement: Supplementary file 7 — Supplementary Figure S7 [file emmm0006-0902-SD7.pdf]

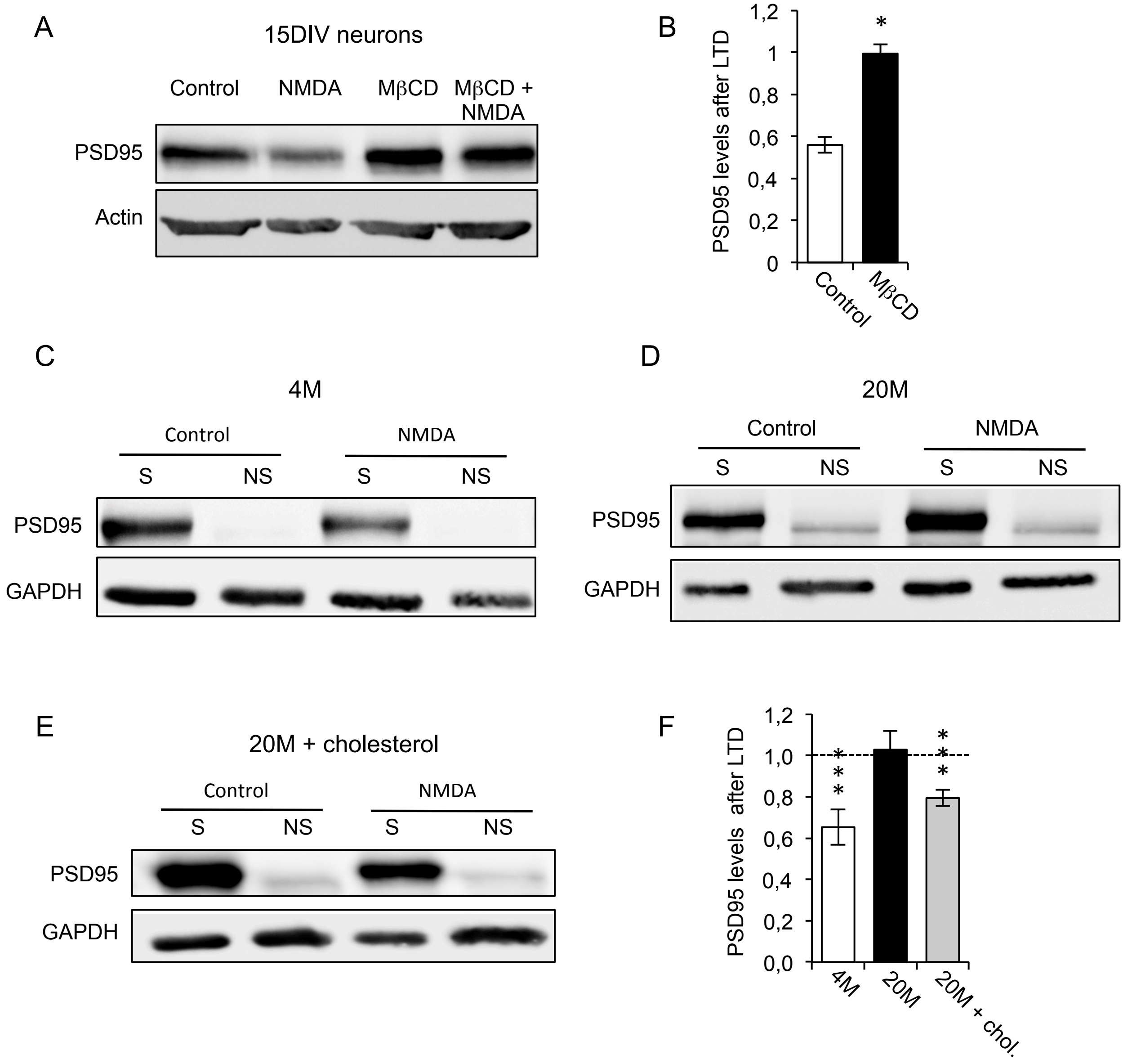

Supplementary Figure S7.

Cholesterol replenishment rescues PSD95 degradation after NMDA-LTD in old hippocampal slices.

A) Western blot showing that cholesterol depletion by MβCD impairs NMDA-LTD induced PSD95 degradation in 15DIV neurons. B) The plot on the right shows the quantification of the remaining PSD95 at 30min after LTD induction in control and MbCD treated neurons (control = 0.56 ± 0.037%, MβCD = 0.995 ± 0.044%, p = 0.0297).

C-D) The blots show the PSD95 levels in synaptosomal (S) and non-synaptosomal (NS) fractions obtained from hippocampal slices in control conditions or after NMDA-LTD induction during 30 min. NMDA-LTD triggers the decrease of PSD95 from the synaptosomal fraction in 4 month-old mice (4M) but not in 20 month-old mice (20M). E) Cholesterol replenishment rescues the NMDA-LTD induced PSD95 degradation in old mice.

F) The plot shows the quantifications of the PSD95 levels remaining after NMDA-LTD. 4M = 0.65 ± 0.087, 20M = 1.03 ± 0.091, 20M + cholesterol = 0.79 ± 0.038 (4M vs 20M: p = 0.034; 20M vs 20M + cholesterol: p = 0.045; 4M vs 20M + cholesterol: p = 0.272).
