# Supplementary material for: Constitutive hippocampal cholesterol loss underlies poor cognition in old rodents
Source: EMBO Mol Med. 2014 May 30;6(7):902–17. doi: 10.15252/emmm.201303711 (PMC4119354; doi:10.15252/emmm.201303711)
Supplement: Supplementary file 8 — Supplementary Table S1 [file emmm0006-0902-SD8.pdf]

Final conc.	Component (FW)	Amount per 1 liter
125 mM	NaCl (58,44)	7,305g / 25 ml of 5M stock
5 mM	KCl (74,56)	0,373g / 5 ml of 1M stock
1,2 mM	NaH2PO4 (137,99)	0,166g / 1,2 ml of 1M stock
1 mM	CaCl2 (147)	0,15 g / 1ml of 1M stock
1,2 mM	MgCl2 (203,3)	10,24g / 1,2 ml of 1M stock
1 uM	ZnCl2	1 ml of 1mM stock
10 mM	Glucose (180,16)	1,8g / 10 ml of 1M stock
25 mM	Hepes (238,31 free acid)	5,96g / 25 ml of 1M stock
0,25%	BSA	2,5 g

Supplementary Table SI.

Modified Hanks medium. The composition of the medium used in the Quantum Dot single molecule tracking experiments is detailed in the table.
