# Supplementary material for: Constitutive hippocampal cholesterol loss underlies poor cognition in old rodents
Source: EMBO Mol Med. 2014 May 30;6(7):902–17. doi: 10.15252/emmm.201303711 (PMC4119354; doi:10.15252/emmm.201303711)
Supplement: Supplementary file 9 — Supplementary Methods [file emmm0006-0902-SD9.pdf]

GluR2 live cell staining for Single Particle Tracking.

GluR2-containing AMPARs were labeled as described in Dahan, M. *et al.* [1]. Neurons were incubated 10 min at 20°C in mouse primary antibody that recognized the extracellular region of GluR2 (2.5 mg/ml in N2 medium plus 20mM Hepes; BD Pharmingen), washed and incubated 10 min at 20°C in biotinylated anti-mouse Fab antibody (2 mg/ml in N2 medium plus 20mM Hepes, Fab/Biotin ratio ~ 1:0.6). Following washes, coverslips were then incubated 1 min at RT in streptavidin-coated QDs emitting at 709 nm (0.2-0.7 nM Invitrogen) in borate buffer (50 mM) supplemented with sucrose (100 mM). After fast rinses, the coverslips were mounted in a custom chamber with preincubated Modified Hank's medium (See Supporting Information Table I). The cells were imaged at 37°C in a closed chamber mounted onto an inverted microscope (Olympus, IXL-UCB) equipped with a 100X objective (NA=1.45, Olympus).

Fab antibody (AffiniPure Fab Fragment, Jackson Immuno Research) was biotinilated using the Biotin-XX Microscale Protein Labeling Kit (Invitrogen). All washes and incubations steps were performed in fresh N2 medium with addition of Hepes buffer (20 mM).

AMPARs tracking and data analysis.

Single molecule tracking was performed with Imaris program. The center of the fluorescence spots was determined with a gaussian fit with a spatial resolution of ~10 nm. Single QDs were identified by the fluorescence intermittency, i.e. the alternation of periods when the QD emitted fluorescence photons and periods when it was dark. Due to these random blinking events, the trajectory of an individual receptor could not necessarily be tracked continuously. Different parts of trajectories were attributed to the same receptor only when the positions of the spot before and after the dark period were compatible with the duration of the extinction

and the typical diffusion coefficient. Diffusion coefficients (*D*) for whole or parts of trajectories were calculated as indicated below.

Trajectory analysis and *D* calculation.

The program used for AMPARs trajectory analysis and *D* calculation [2] is accessible at VIB Bioinformatics Training and service facility (BITS) portal

http://www.bits.vib.be/index.php?option=com_content&view=article&id=17203982:menchon-tools&catid=24:developed-at-vib&Itemid=504

Synapse staining

In control experiments synapses were labeled by incubation for 1-2 min at 20°C with 1 nM Mitotracker (Green-Fluorescent Mitotracker, Molecular Probes, Leiden, The Netherlands) before GluR2 labeling as previously described [3].

In these experiments a picture of the stained synapses was taken immediately before start recording the sets of 1000 images for GluR2 tracking. The centers of the green fluorescence spots corresponding to synapses were determined by a gaussian fit and the coordinates for every center were obtained using the program Imaris. We considered that a confinement site was at a synapse when the distance between the center of the confined area and the center of a synapse was < 1.3 μm. In our experiments 47-48% of the confinement sites identified were coincident with synapses. The *Di* obtained using the synaptic confinement sites identified by this method were similar to the *Di* obtained for the all the confinement sites. For example, in 15DIV neurons, the *D* value for confinement sites overlapping synapses was $Di = 0.0282 \pm 0.0064$ $\mu m^2/s$ whereas the *D* value for all the confinement sites was $Di = 0.0263 \pm 0.0078$ $\mu m^2/s$ ($p>0.05$, n = 359 trajectories). When the cells were stimulated with glutamate the

values obtained for confinement sites overlapping synapses were: *Di* = 0.0382 ± 0.0090 $\mu m^2/s$ and for all the confinement sites: *Di* = 0.0339 ± 0.0034 $\mu m^2/s$ ($p>0.05$, n = 295 trajectories). Thus, we assumed that the *D* values obtained for confinement sites correspond to the *D* values for synaptic AMPARs.

In individual AMPAR tracking experiments the diffusion coefficients calculated from all the trajectories analyzed (ranging from 250 to 500) have a one-tail distribution. The effect of glutamate in single experiments has been studied using non-parametric Mann-Whitney tests. The mean values of the medians follow a normal distribution and thus groups of experiments were compared using unpaired t test.

Repeated ANOVA tests were used to evaluate the data in all the electrophysiology and behavioral experiments.

References.

1. Dahan M, Levi S, Luccardini C, Rostaing P, Riveau B, et al. (2003) Diffusion dynamics of glycine receptors revealed by single-quantum dot tracking. Science 302: 442-445.

2. Menchon SA, Martin MG, Dotti CG APM_GUI: analyzing particle movement on the cell membrane and determining confinement. BMC Biophys 5: 4.

3. Groc L, Heine M, Cognet L, Brickley K, Stephenson FA, et al. (2004) Differential activity-dependent regulation of the lateral mobilities of AMPA and NMDA receptors. Nat Neurosci 7: 695-696.
