# Supplementary figures and images for: Constitutive hippocampal cholesterol loss underlies poor cognition in old rodents
Source: EMBO Mol Med. 2014 May 30;6(7):902–17. doi: 10.15252/emmm.201303711 (PMC4119354; doi:10.15252/emmm.201303711)

Supporting Information Figure 1

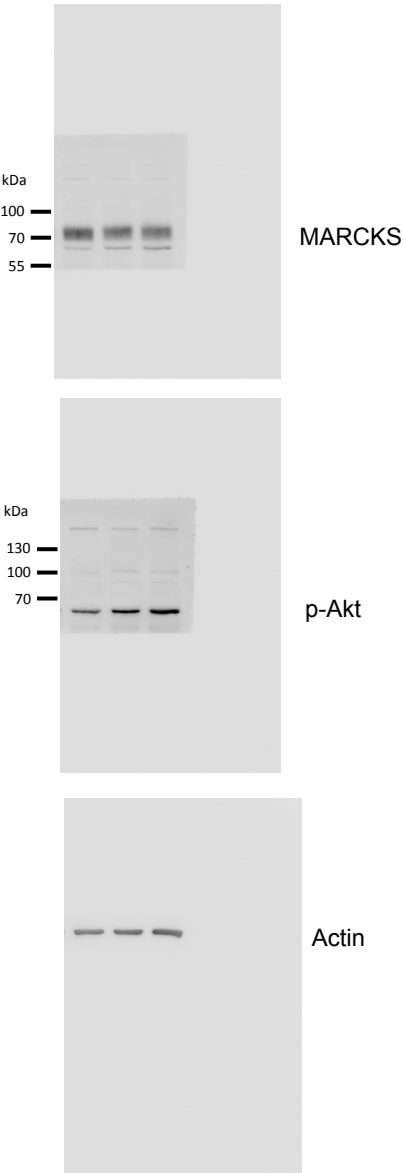

Supplement: Supplementary file 10 — Source Data for Supplementary Figure S1 [file emmm0006-0902-SD10.pdf]

Figure 1

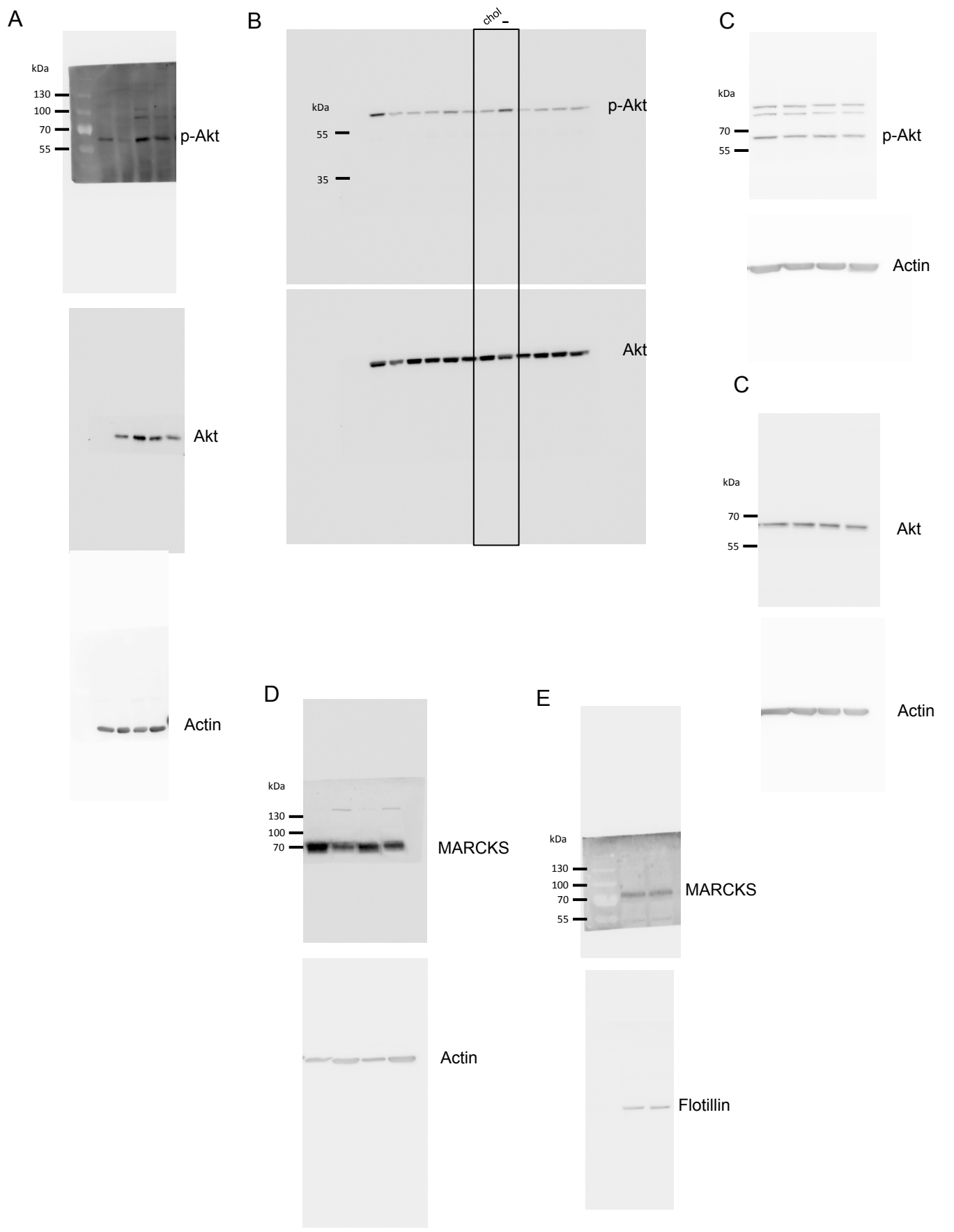

Supplement: Supplementary file 12 — Source Data for Figure 1 [file emmm0006-0902-SD12.pdf]

Figure 2

A

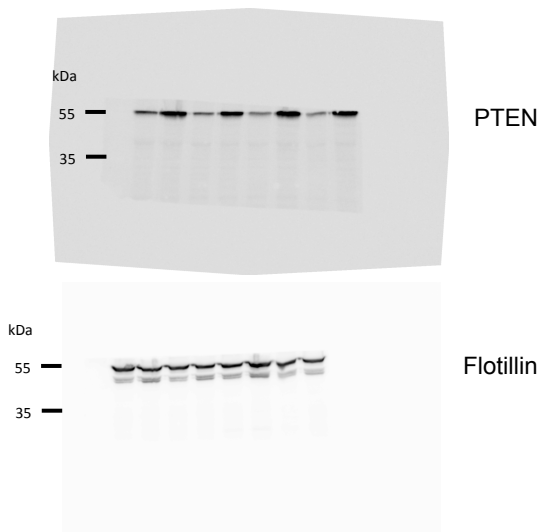

B

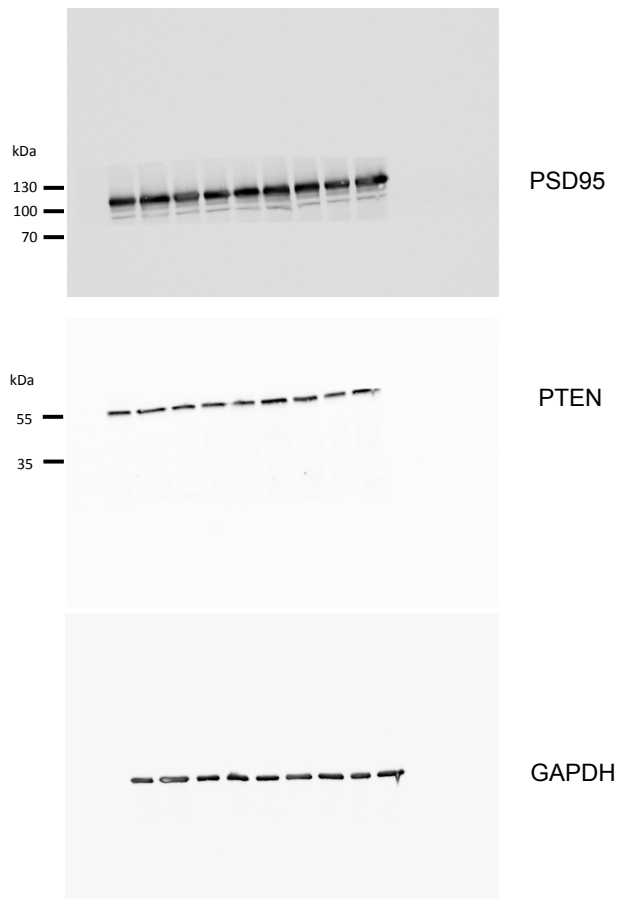

Supplement: Supplementary file 13 — Source Data for Figure 2 [file emmm0006-0902-SD13.pdf]
